# Knowledge Construction and Co-Construction in the Era of Team-Based Care: A Scoping Review

**DOI:** 10.1007/s40670-026-02896-0

**Published:** 2026-10-12

**Authors:** Pui Yin Jason Cheung, Qing He, Yumeng Yue, Xiaoai Shen, John Ian Wilzon T. Dizon, George Lim Tipoe, Fraide A. Ganotice

**Affiliations:** 1 Department of Orthopaedics and Traumatology, Li Ka Shing Faculty of Medicine, The University of Hong Kong, (Administration Office, 5/F, Professorial Block, Queen Mary Hospital), Pok Fu Lam, Hong Kong; 2 Bau Institute of Medical and Health Sciences Education, Li Ka Shing Faculty of Medicine, The University of Hong Kong, (5/F William MW Mong Block, Faculty of Medicine Building, 21 Sassoon Road), Pokfulam, Hong Kong

**Keywords:** Knowledge Construction, Knowledge Co-Construction, Medical Education, Scoping Review

## Abstract

**Background:**

Knowledge construction and co-construction are central to preparing health professions students for collaborative, team-based care. However, evidence on how these processes are conceptualised, supported, and studied in health professions education remains dispersed.

**Methods:**

This scoping review was conducted in accordance with JBI guidance for scoping reviews and reported using the PRISMA-ScR checklist. Arksey and O’Malley’s framework informed the stages of identifying the research question, searching for relevant studies, selecting studies, charting data, and collating and summarising findings. PubMed, Scopus, Web of Science, ERIC, and PsycINFO were searched for peer-reviewed empirical studies published before June 2025. After duplicate removal, 2,311 records were screened, 274 full texts were assessed, and 20 studies were included.

**Results:**

Studies covered medicine, nursing, and pharmacy students. Key facilitators included structured collaborative modalities, active learning strategies, and technological tools. Challenges comprised time constraints, unequal student participation, technical barriers, limited digital literacy, and divergent clinical reasoning perspectives.

**Conclusions:**

Knowledge construction and co-construction in health professions education are supported by intentional pedagogical, technological, and relational scaffolding. Future research should more clearly operationalise these constructs, examine interprofessional contexts, and develop strategies to address participation imbalances, time pressures, and inequitable access to learning opportunities.

## Background

Health systems increasingly recognize that high-quality care depends on collaborative, team-based practice, in which individuals from diverse disciplines collaborate with patients, families, and communities to deliver the highest quality of care across settings [1]. Achieving this level of practice requires the dismantling of disciplinary silos and the development of collective intelligence in healthcare. Grant [2] argues that “the focus on independent achievement leaves many students woefully unprepared for an interdependent world of work,” underscoring a persistent gap between what is needed in practice and how students are educated.

Collaborative problem-solving activities are now widely promoted in health professions education as a means of closing this gap. These activities enable learners to co-construct knowledge, integrate diverse perspectives, and develop the critical thinking and communication skills essential for safe, coordinated care [3].

However, research on knowledge construction and co-construction in these settings is dispersed across disciplines, contexts, and methodologies, with limited systematic synthesis. A scoping review is therefore warranted to map the existing empirical literature, identify facilitating factors (contextual or instructional factors that actively promote or ease the collaborative learning process), barriers (structural, psychological, or logistical obstacles that hinder student interaction), tools, and theoretical underpinnings, and clarify how learners construct and co-construct knowledge both among peers and between students and teachers. By providing a comprehensive overview, this review seeks to inform the design of future educational interventions, guide curriculum development, and bridge key gaps in knowledge-construction research within health professions education.

### Knowledge Construction and Knowledge Co-Construction

Knowledge construction (KC) involves the creation of new knowledge or understanding through personal interpretation and integration of existing knowledge and experience [4]. It involves actively engaging with information, critically analyzing and evaluating it, making connections to prior knowledge, and constructing new meanings or knowledge based on those connections. Knowledge construction involves a range of cognitive processes, including interpreting and evaluating new information, sharing, and criticizing ideas at different levels. This process is often influenced by personal experiences, perspectives, and cultural backgrounds as individuals construct their own unique understanding of the world [4]. Knowledge sharing is the exchange of existing knowledge through interaction between different individuals. However, knowledge construction is an ongoing, dynamic process that occurs through a variety of activities, such as reading, researching, reflecting, discussing, and applying knowledge in the real world [5].

Knowledge co-construction (KCC), on the other hand, is the process of building knowledge collaboratively with others (e.g., in a group or team). It involves individuals working together to share and integrate their personal knowledge and experiences to create new knowledge that is collectively owned and shared [6]. Gunawardena et al.‘s five-stage Interaction Analysis Model (IAM) of knowledge co-construction is grounded in social constructivist theory. The five stages of the IAM model are (I) information sharing and comparison, (II) discovery and exploration of dissonance or incongruence, (III) negotiation of meanings or co-construction of knowledge, (IV) testing of proposed syntheses or co-constructions, and (V) presentation of summary of agreement, application of new knowledge, or metacognitive statements [7]. This process requires the active participation and engagement of all members as they contribute their unique perspectives and expertise to the team’s shared understanding. Knowledge co-construction is commonly used in educational and professional settings where students co-construct knowledge as well as work in teams to solve complex problems and make decisions based on shared knowledge through group projects, discussions, and other collaborative activities [8].

### Importance of Knowledge Construction and Knowledge Co-Construction in Health Professions Education

Over the past decade, there have been significant changes in curricular approaches to health professions education (HPE). Many medical professionals and educators have realized that traditional approaches are insufficient to develop the cognitive and collaborative skills required of healthcare professionals [9]. Knowledge construction and knowledge co-construction can help promote active learning, which previous research has demonstrated to be more effective than passive learning methods such as listening or reading. When learners are actively engaged with the material, they are more likely to understand and retain information and develop critical thinking and problem-solving skills [10]. When learners are actively engaged with material via these constructivist mechanisms, they demonstrate a higher likelihood of deep conceptual understanding, long-term information retention, and advanced development of critical thinking and collaborative problem-solving skills [11]. Medical knowledge does not exist in isolation and ultimately needs to be utilized in clinical practice. Therefore, in medical education, educators have reformed the use of different pedagogical approaches to help simulate clinical practice settings to help students learn and train skills [12]. In this process, knowledge construction and knowledge co-construction enable learners to relate new information to their existing knowledge and experience, apply their knowledge to real-life scenarios, and understand the practical relevance of what they are learning [8]. This contextualized learning promotes better integration of theoretical knowledge with clinical practice.

Ultimately, medical education aims to improve patient care outcomes. In medical practice, healthcare professionals must collaborate and work as a team to provide optimal patient care. Knowledge co-construction in medical education simulates this collaborative environment and prepares learners for future interprofessional collaboration. By co-constructing knowledge, learners develop effective clinical practice skills, communication, and teamwork skills, which are critical in healthcare settings [13] .

### Rationale and Objective for the Current Review

This review aims to clarify how students construct and co-construct knowledge in health professions education and to identify contextual triggers that support these processes. As healthcare education increasingly emphasises active and collaborative learning, and as technological advances and shifts from professional to interprofessional care transform the healthcare landscape, it is essential to understand knowledge co-construction processes in health professions education. A scoping review methodology was selected to map the key concepts, types of evidence, and gaps in this broad, emerging, and heterogeneous body of literature [14]. This scoping review seeks to identify best practices and areas for improvement in the use of knowledge construction and co-construction in health professions education.

The overarching objective is to understand how educators promote students’ knowledge construction and knowledge co-construction in health professions education. To address this objective, we posed the following research questions:

- RQ1: What teaching strategies and pedagogical interventions are commonly utilized by educators to support knowledge construction and co-construction?
- RQ2: What contextual, instructional, or relational factors facilitate effective knowledge construction and co-construction in health professions education settings?
- RQ3: What core challenges do educators and students encounter during the process of knowledge construction and co-construction in health professions education?

## Methods

The process of conducting the scoping review was guided by the PRISMA guidelines [15] and informed by Arksey and O’Malley’s framework [16].

### Search Strategy

We sought to identify studies on knowledge construction and co-construction in health professions education. A comprehensive search was conducted in five databases: PubMed, Scopus, Web of Science, ERIC, and PsycINFO. A combination of keywords was used to capture the concepts of knowledge construction, knowledge co-construction, and health professions education: (“knowledge construction” OR “constructivist learning” OR “knowledge co-construction”) AND (“health professions education” OR “medical education”). Search strings were adapted for each database. The search was limited to peer-reviewed articles published before June 2025.

### Eligibility Criteria

Eligibility criteria were established using the Population, Concept, and Context (PCC) framework to guide a comprehensive and systematic scoping review. Studies were included if they: (1) focused on students, educators, or active participants within HPE, including medicine, nursing, pharmacy, dentistry, and allied health fields; (2) explicitly investigated the processes, mechanisms, or outcomes of knowledge construction or knowledge co-construction within an educational framework; (3) were conducted in formal or informal HPE learning environments, including university classrooms, simulated settings, clinical training units, or digital/online platforms; and (4) comprised empirical research (qualitative, quantitative, or mixed-methods) published as peer-reviewed English-language journal articles.

Conversely, studies were excluded if they: (a) lacked an explicit educational framework, focusing instead on clinical workplace metrics, routine patient care, or professional practice without a learner-centered instructional component; (b) were situated in K–12 schooling, or non-health disciplines; (c) examined generic active learning or group work without explicitly operationalizing or evaluating the cognitive stages of knowledge construction or co-construction; or (d) other literature, including dissertations, book chapters, conference abstracts, editorials, opinion pieces, or non-scholarly commentaries lacking reproducible methodology or peer-reviewed vetting.

### Study Selection

The study selection process followed the PRISMA guidelines [15]. Three authors independently screened titles and abstracts of all retrieved records and classified them as “include”, “exclude”, or “uncertain”. To ensure a comprehensive capture of relevant literature, any record marked as “uncertain” by a reviewer automatically advanced to the full-text review stage. After removing duplicates, 2,311 articles were screened against the predefined eligibility criteria (publication date, language, study design, and relevance to the research questions). During the full-text assessment, any disagreements regarding study eligibility between the two reviewers were resolved through an iterative consensus process. If a bilateral agreement could not be reached, a third senior author reviewed the text independently and made the final decision. Following full-text assessment, 20 studies met the criteria and were included in the review.

### Data Analysis and Coding

We used content analysis to code the selected papers [17, 18]. A priori coding schema was developed based on the research questions, comprising five primary categories: (a) study design, (b) educational setting (e.g., undergraduate, postgraduate, continuing professional development), (c) disciplinary field (e.g., medicine, nursing, allied health), (d) sample characteristics (including sample size and participant demographics), and (e) primary intervention/outcome measures. Data extraction was performed independently by one reviewer using the a priori coding schema. A second reviewer independently verified 100% of the extracted data against the source articles to ensure accuracy and consistency. Disagreements were resolved through consensus discussion; where consensus could not be reached, a third reviewer arbitrated.

### Quality Assessment

Consistent with scoping review methodology, we did not conduct a formal risk-of-bias assessment [15]. However, all included studies were appraised for methodological clarity and relevance to the review questions through independent review by three authors. The appraisal focused on the alignment between study design, research questions, and reported outcomes. Disagreements were resolved through discussion. No studies were excluded based on quality appraisal, as the objective was to map the breadth of evidence rather than to synthesize effect sizes.

## Results

### Study Selection

**Fig. 1:**
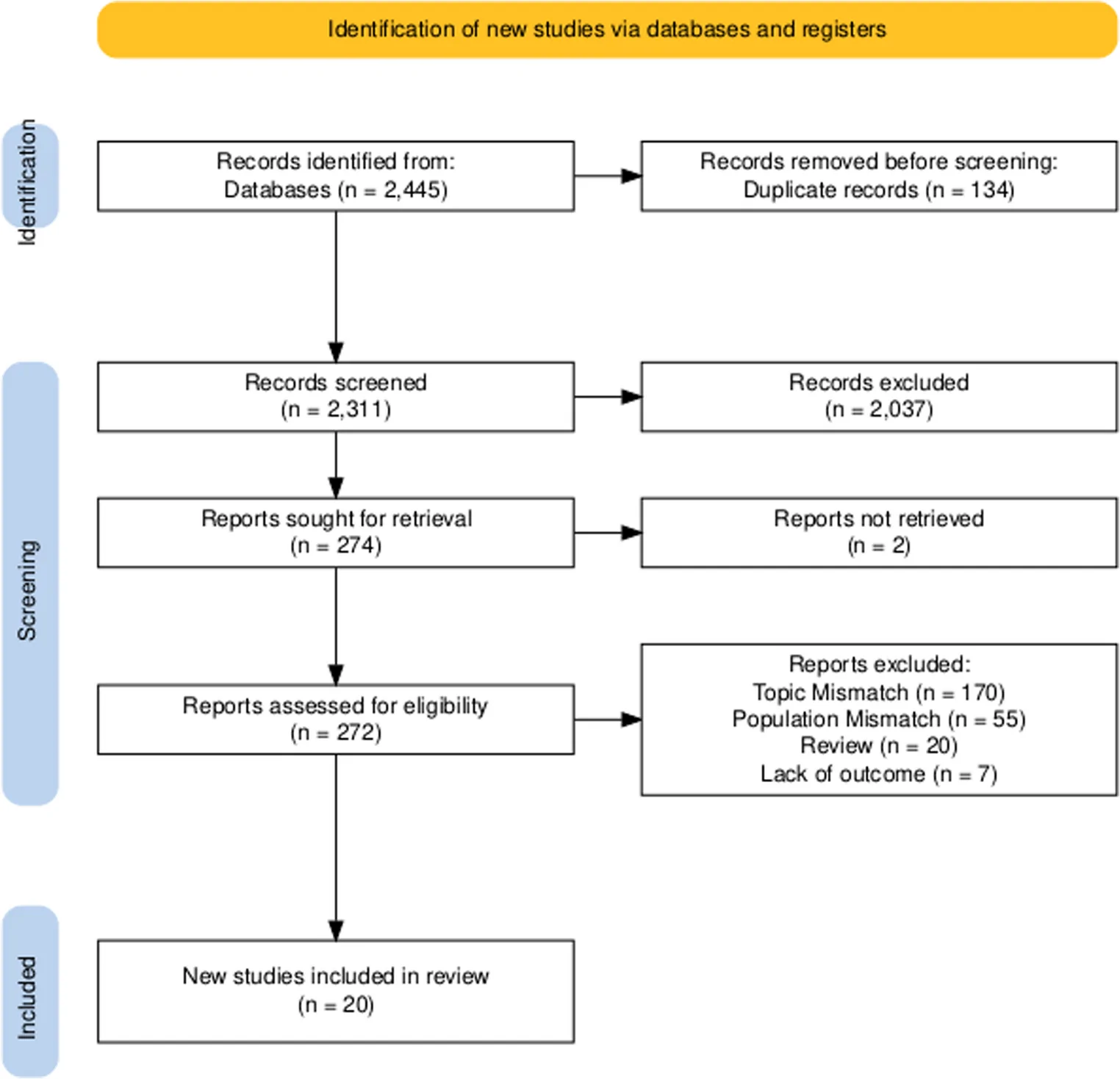
The flowchart of searching and filtering process

The database search strategy identified 2445 records. After removing 134 duplicates, 2,311 records title and abstract screening. Of these, 2037 were excluded, leaving 274 full-text articles for assessment. Twenty studies [19–38] met the eligibility criteria and were included in the review (Fig. 1).

### Study Characteristics

From all the included studies (Table 1), four studies (20%) were conducted in Hong Kong, while two studies were conducted in each of the following: Belgium, China, the Netherlands, and the USA (10% each). Additionally, one study involved students from both Hong Kong and Canada, and another included student from both the UK and Malaysia.

**Table 1:** Summary of articles reviewed

First Author and Year	Region	Discipline	Sample Size	Duration	KC and KCC Focus	Statistical Methods
Floren, L. C. (2020)	USA	Medicine, Pharmacy	*n* = 32	Four weeks to two semesters	KC	Mixed Methods
Wang, M. (2013)	Hong Kong	Medicine	*n* = 35	NR	KC	Mixed Methods
Loyens, S.M.M (2006)	The Netherlands	Clinical Psychology	*n* = 293	NR	KC and KCC	Quan
da Silva, A. P. S. (2010)	Brazil	Nursing	*n* = 185	NR	KC	Qual
Wu, B. (2012)	Hong Kong	Medicine	*n* = 29	NR	KC	Mixed Methods
Bridges, S. M. (2009)	Hong Kong	Dentistry	*n* = 52	NR	KC	Qual
Weurlander, M. (2009)	Sweden	Medicine	*n =* 53	NR	KC and KCC	Qual
De Wever, B. (2008)	Belgium	Medicine	*n* = 49	One month	KCC	Qual
Lee, L. (2017)	Canada and Hong-Kong	Medicine	*n* = 4	Five days	KC	Mixed Methods
Timmers, S. (2008)	Belgium	Pharmacy	*n* = 77	Each case two week	KC	Qual
De Leng, B. (2015)	The Netherlands	Medicine	*n* = 70	NR	KC	Mixed Methods
Lestari, E. (2023)	Indonesia	Medicine, Nursing and Midwifery	*n* = 476	Six weekdays (28 h)	KC	Mixed Methods
Zheng, B. (2023)	Hong Kong	Medicine	*n* = 24	Two hours	KC	Qual
Heng, W. (2025)	UK and Malaysia	Medicine	*n* = 291	NR	KC	Mixed Methods
Bland, T. (2025)	USA	Medicine	*n* = 40	NR	KC	Quan
Afshar, M. (2024)	Iran	Medicine	*n* = 210	NR	KC	Quan
Wang, Y. (2024)	China	Nursing	*n* = 185	Two years	KC	Mixed Methods
Lin, W. (2025)	China	Chinese Medicine	*n* = 250	NR	KC	Quan
Ross, A. J. (2024)	South Africa	Medicine	*n* = 24	Seven weeks	KCC	Qual
García-Expósito, J. (2025)	Spain	Nursing	*n* = 137	Three weeks	KC	Qual

*NR*: Not reported, *KC*: Knowledge construction, *KCC*: Knowledge co-construction, *Quan*: Quantitative, *Qual*: Qualitative

The studies spanned several disciplines, with the majority of participants from medicine (13 studies, 65%). Other disciplines included nursing (3, 15%), pharmacy (2, 10%), Chinese medicine (1, 5%), clinical psychology (1, 5%), dentistry (1, 5%), and one multidisciplinary study including medicine, nursing, and midwifery. Some studies also involved teachers as stakeholders in the learning process. These included tutors or facilitators (3, 15%), healthcare professionals (2, 10%), and professors or other faculty staff (1, 5%). Healthcare professionals were typically aligned with the students’ disciplines or were domain experts, such as physicians and pharmacists [19].

Study duration varied widely, from a single two-hour session [31] to a two-year intervention [35]; many studies did not specify duration (8, 40%). Sample sizes ranged from very small (e.g., *n* = 4 in [27]) to relatively large (e.g., *n* = 476 in [30]). Most studies (16, 80%) focused on knowledge construction, whereas a few examined knowledge co-construction alone (2, 10%) or in combination with knowledge construction (2, 10%).

### Methodological Approaches

Studies used different outcome measurements to evaluate the effectiveness and usability of teaching tools and teaching methods. Eight studies (40%) used both quantitative and qualitative methods as part of the mixed methods to analyze the results, and many studies (8, 40%) use qualitative methods to explore the processes and experiences of knowledge construction and co-construction. Only four (20%) studies employ quantitative methods to measure and analyze data related to knowledge construction and co-construction. The outcomes to evaluate the results included surveys of students’ perceptions of teaching tools, scores, knowledge construction level, and the content of student-teacher/ student-student interactions that occur during the lesson. Some studies also included focus group discussions to analyze the specific perceptions or attitudes of students and teachers.

### Education Context

Problem-Based Learning (PBL, a teaching method that small groups of learners collaborate to analyze clinical scenarios) was the most prevalent educational strategy, with 20% of the studies reporting its use. Case-Based Learning (CBL, a teaching method that uses authentic patient scenarios to contextualize theoretical knowledge) was the second most common contexts, with 15% of the studies. Other strategies included Interprofessional Education (IPE, a teaching method that involve students from two or more health and social care professions), traditional classroom settings, clinical rotations, active learning, flipped teaching, peer-assisted teaching, BOPPPS Model (6-part teaching approach stands for bridge-in, outcomes, pre-assessment, participatory learning, post-assessment, and summary), and innovative approaches such as AI integrated with Cinematic Clinical Narratives (CCNs) or Moxibustion Robots.

### Theoretical Frameworks

Various frameworks used in knowledge construction and knowledge co-construction studies in health professions education have been identified. These include Gunawardena’s knowledge construction framework [19], the cognitive apprenticeship model [20], Paulo Freire’s framework [22], cognitive theories and models [39], the social constructivist theory [40], the Chronometric Constructive Cognitive Learning Evaluation Model [41], Hadwin’s framework [42], Veerman and Veldhuis-Diermanse’s framework [43], and the framework proposed by Weinberger and Fischer [44] to analyze argumentative Knowledge construction in computer-supported collaborative learning. Additionally, there is a proposed model and process to redesign and deliver the syllabus effectively, integrating PBL with active learning strategies, as well as an online case-based Learning model [30]. These frameworks provide theoretical underpinnings and guidance for understanding and facilitating knowledge construction and knowledge co-construction in health professions education contexts.

### Facilitator of Knowledge Construction and Co-Construction

**Fig. 2:**
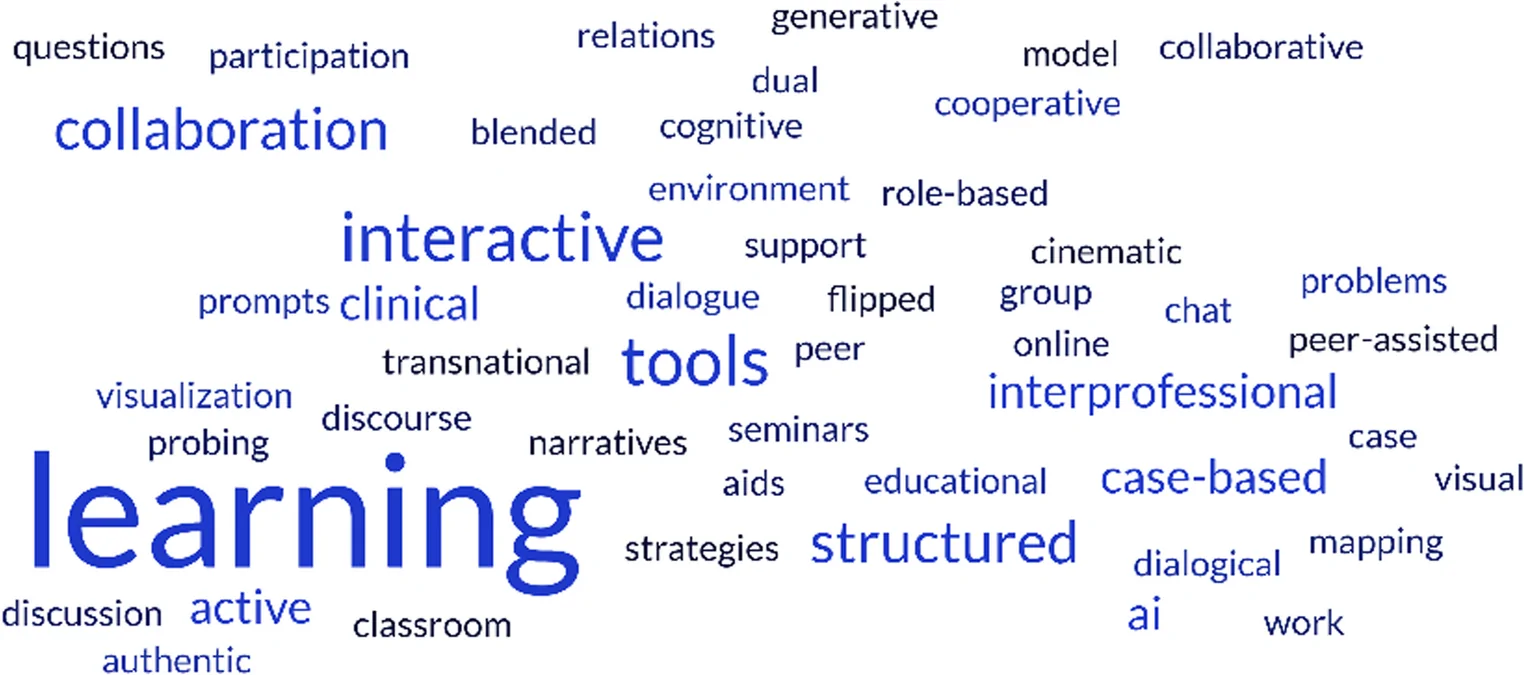
Word Cloud of Facilitators Reported in The Included Studies

The review identified several key facilitators that enhance knowledge construction and co-construction. These facilitators were grouped into four main categories: pedagogical strategies, collaborative learning environments, technological tools, and contextual relevance (Fig. 2). An analytical assessment of the term distribution in word cloud of Fig. 2 reveals a thematic structure. “Learning” is the conceptual anchor, closely surrounded by operational descriptors such as “interactive” and “participation”, indicating a dominant active-learning paradigm. A secondary cluster comprising “collaboration”, “interprofessional”, and “team-based” suggests that structured group dynamics and interprofessional contexts are primary vehicles for co-construction. The prominence of “tools”, “blended”, “online”, and “AI” further indicates the increasing integration of technology in facilitating collaborative learning. Collectively, this distribution positions effective knowledge construction as dependent on interactive, structured, technology-enhanced, and interprofessionally collaborative systems.

#### **Pedagogical Strategies**

Pedagogical strategies are important approaches for supporting knowledge co-construction. Structured dialogue and questioning [19, 31] offered by educators encouraged active participation. Furthermore, when educator ask probing questions, students can deepen their critical thinking. PBL/CBL [21, 30, 35] engaged learners in real-world scenarios, fostering practical application and teamwork. Blended and flipped learning models [24, 34] offered a flexible and comprehensive learning experience and adapted to different learning preferences.

#### **Collaborative Learning Environments**

Creating collaborative learning environments can significantly influence knowledge construction and knowledge co-construction by fostering interaction and teamwork among students. These environments provide a platform for groups of students to collaborate on problem-solving, task completion, or product creation [45]. Collaborative environments including interprofessional teamwork, peer-assisted activities and group-based learning. Interprofessional education [19, 30] leveraged diverse perspectives to enrich understanding. Peer-assisted and cooperative learning [22, 34] promoted autonomy and mutual support. Role-based and group learning [24, 26] strengthened shared accountability, though group dynamics occasionally posed challenges. By engaging in collaborative knowledge-building practices within these environments, students can enhance their comprehension, critical thinking, and problem-solving abilities, ultimately contributing to a richer and more comprehensive understanding of the subject matter at hand.

#### **Contextual and Active Learning**

Active learning and authentic strategies encourage students to apply theoretical knowledge to real-life scenarios and promote deeper understanding and construction of new knowledge. Clinical and rural immersion [37] linked learning to real-world settings, while cinematic narratives [33] increased engagement. Autonomous and transnational learning [17, 32] encouraged self-direction and cross-cultural perspectives. By encouraging students to take an active role in their learning, engage with course material in a hands-on manner, and interact with their peers, educators can facilitate a more dynamic and enriching learning experience that promotes deeper understanding and the construction of new knowledge.

#### **Technology-Enhanced Learning**

Technology tools such as online platforms, virtual simulations, multimedia resources and Gen AI provide students with opportunities for interactive learning, self-directed exploratory inquiry, and ongoing access to a wide range of information sources that enable students to construct and co-construct knowledge collaboratively. Visualization aids [20, 29] simplified complex concepts through diagrams and dual mapping [23] to enhance understanding of complex concepts. Interactive platforms [28, 38], including educational chats and forums, facilitated asynchronous critical dialogue. Advanced tools including generative AI [33, 36] offered personalized learning tools for students to build knowledge into progressively more complex and systematic structures.

### Challenges in Implementing Knowledge Construction and Co-Construction

Implementing knowledge construction and co-construction in educational settings faces several technological challenges. Frequent problems with online platforms and software or hardware failures can hinder students’ communication and disrupt the learning process [19, 24, 27, 32, 34, 35]. Limited access to necessary technologies, such as AI tools and other cognitive tools, particularly in resource-constrained environments, is another important barrier [28, 32, 36, 37]. Moreover, technology-enhanced learning often depends on a variety of personal devices (computers, mobile phones, tablets) and operating systems, leading to compatibility issues that make it difficult for students and teachers to access and use these tools effectively.

Time constraints were significantly highlighted in the included studies. Limited interaction time often affected the depth of knowledge construction, while insufficient preparation time negatively impacting learning quality [19, 35]. Balancing collaborative learning activities with other responsibilities, such as internships or other courses, can be particularly challenging for students [26, 28, 30, 35].

Motivation and engagement issues are prevalent, especially among students in a traditional curriculum who may lack the incentive to participate in constructivist activities [21, 22]. This uneven distribution of involvement can hinder equitable participation, engagement, and knowledge construction outcomes in collaborative learning settings. Limited technological literacy, including difficulties with educational chat, advanced AI tools, and even basic computer skills, further hinders effective participation in online forums and digital learning environments [22, 32, 38].

Isolation and lack of interaction are common drawbacks of online learning. In a traditional classroom, there are many opportunities for social interaction with classmates and the instructor. In medical education, active engagement in teaching skills is greatly facilitated by face-to-face interaction, as it encourages direct contact and communication between students and both their peers and instructors. In Lestari et al. study [30], students expressed a desire to learn together offline to establish a stronger bond of cooperation, indicating the limitations of online collaborative learning models. Bridges et al. [24] also reported similar findings, noting that group dynamics and limited face-to-face interaction can affect team cohesion, collaboration, and knowledge construction. In rural or isolated settings, geographical barriers may also limit access to peer support and consistent teaching quality, complicating the implementation of knowledge construction and co-construction [37].

## Discussion

### Current State and Scope of KC/KCC Research in HPE

This scoping review investigates the existing research on knowledge construction and co-construction in healthcare education, covering the period from 2006 to 2025. Overall, the empirical literature describing knowledge construction and co-construction in medical education remains very limited. The studies were conducted across Asia, Africa, Europe, North America, and South America. These studies generally adopted mixed methods designs, incorporating qualitative and quantitative analyses.

The included studies primarily focused on students from medicine, nursing, and pharmacy. This suggests that KC/KCC is often examined within single-discipline courses, with relatively few interprofessional contexts. Given increasing calls from major healthcare organizations, such as the WHO [46] and the Institute of Medicine [47], future training initiatives should prioritize interprofessional KC/KCC to better prepare students for the realities of modern healthcare practice. Furthermore, involving a more diverse range of healthcare students will be important to enhance the generalizability of KC/KCC implementation across health professions education.

### Facilitators and Challenges Shaping KC/KCC Implementation

Pedagogical strategies, such as structured dialogue, PBL/CBL, and blended learning models, were found to be highly effective in promoting active participation and critical thinking to facilitate KC/KCC. These findings provide valuable insights into the factors that enable KC and KCC in health professions education and can guide the development of effective teaching practices and curricula in this field.

PBL and CBL emerged as the most prevalent strategies, aligning closely with key facilitators such as structured dialogue, real-world relevance, and collaborative learning. This approach is favored in medical education due to its ability to facilitate active learning, critical thinking, clinical relevance, teamwork, and lifelong learning skills—all vital for preparing healthcare professionals [48] showing these educational strategies are beneficial for long-term retention and skill development in medical education. Future research may, therefore, explore the areas that have been under-investigated. For example, knowledge construction and co-construction may be explored in the context of interprofessional education to understand how divergent ideas from members of diverse disciplines converge towards a shared understanding. Consistent with community of inquiry [49], the use of these active learning pedagogies promoted social presence and cognitive presence.

Despite the identified facilitators, several challenges hinder the effective implementation of KC and KCC. The reviewed articles revealed a range of challenges identified in the literature. These challenges included technological issues, time constraints, motivation and engagement problems, limited technological literacy and the isolation in online learning. These findings shed light on the multifaceted challenges that must be addressed to facilitate KC and KCC in health professions education effectively. These barriers mirror challenges identified in wider edTech studies, such as the ‘digital divide’ in healthcare education [50] and cognitive overload risks in multimedia learning [51]. Our review extends these findings by adding on AI-specific literacy gaps (e.g [47]). As edTech is developing, future research should also focus on developing strategies and interventions to overcome these challenges and create conducive environments for successful KC and KCC.

### Synthesis and Implications for Practice

In summary, these findings demonstrate that while pedagogical and technological scaffolds effectively support collaborative knowledge construction, the field remains fragmented by disciplinary silos, geographical dispersion, and theoretical diversity. For educators, the evidence strongly supports integrating PBL, CBL, and other active learning strategies to enhance KC and KCC, while simultaneously addressing technological barriers through targeted training and infrastructure investment. Mixed methods designs and explicit theoretical frameworks can make future studies easier to compare and replicate. For researchers, future work should prioritize: (a) exploring KC/KCC in interprofessional and under-investigated disciplinary contexts; (b) investigating the long-term impact of these approaches on student learning outcomes and professional development; and (c) developing standardized, validated instruments to measure KC/KCC processes across diverse educational settings. Only through such intentional, multi-pronged efforts can the field move from its current seminal phase toward a mature, evidence-based body of knowledge capable of informing curriculum design and healthcare workforce preparation at scale.

### Limitations

This review has several limitations. First, we restricted inclusion to English-language studies. This may have led us to overlook relevant work conducted in other languages and biased our synthesis toward Anglophone contexts, thereby limiting the global generalisability of our findings. Second, although we applied explicit working definitions of knowledge construction and knowledge co-construction, the inconsistent and sometimes interchangeable use of these terms in the included studies constrained the precision with which we could classify them. As a result, some nuances in how knowledge construction and co-construction operate across contexts may not be fully captured in this review.

## Conclusion

This scoping review examined educational initiatives in health professions education that employ knowledge construction and co-construction and offers guidance to educators seeking to advance scholarship and practice in this area. Our findings highlight that while structured pedagogical strategies such as CBL and PBL strongly facilitate the process, their success is heavily augmented by interactive digital platforms and emerging technologies like generative AI. However, these facilitators do not operate in a vacuum; they are consistently constrained by systemic challenges, including student time limitations, participation imbalances, and divergent clinical reasoning styles.

The implications of these findings are twofold. First, medical science educators should move beyond simply implementing group work; they must strategically design curricula that build in structural buffers for time and explicitly scaffold equitable student participation. Second, as healthcare shifts toward a more integrated model, future research must move past single-discipline environments. There is an urgent need for rigorous, empirical studies that investigate how KC and KCC operate within complex interprofessional frameworks and digitally mediated, asynchronous learning environments. Addressing these research gaps and applying these instructional designs will ultimately prepare future healthcare professionals to transition seamlessly into high-functioning, collaborative clinical teams, thereby enhancing patient safety and care quality.
